# Non-Destructive Spectroscopic and Imaging Techniques for the Detection of Processed Meat Fraud

**DOI:** 10.3390/foods10020448

**Published:** 2021-02-18

**Authors:** Kiah Edwards, Marena Manley, Louwrens C. Hoffman, Paul J. Williams

**Affiliations:** 1 Department of Food Science, Stellenbosch University, Private Bag X1, Matieland, Stellenbosch 7602, South Africa; 17620759@sun.ac.za (K.E.); mman@sun.ac.za (M.M.); 2 Department of Animal Sciences, Stellenbosch University, Private Bag X1, Matieland, Stellenbosch 7602, South Africa; lch@sun.ac.za or; 3 Centre for Nutrition and Food Sciences, Queensland Alliance for Agriculture and Food Innovation (QAAFI), The University of Queensland, Health and Food Sciences Precinct, 39 Kessels Rd, Coopers Plains 4108, Australia

**Keywords:** meat fraud, processed meat, non-destructive techniques, real-time monitoring

## Abstract

In recent years, meat authenticity awareness has increased and, in the fight to combat meat fraud, various analytical methods have been proposed and subsequently evaluated. Although these methods have shown the potential to detect low levels of adulteration with high reliability, they are destructive, time-consuming, labour-intensive, and expensive. Therefore, rendering them inappropriate for rapid analysis and early detection, particularly under the fast-paced production and processing environment of the meat industry. However, modern analytical methods could improve this process as the food industry moves towards methods that are non-destructive, non-invasive, simple, and on-line. This review investigates the feasibility of different non-destructive techniques used for processed meat authentication which could provide the meat industry with reliable and accurate real-time monitoring, in the near future.

## 1. Introduction

With the rise in demand to produce food for the ever-growing population, there is a considerable economic gain in adulterating foods. In addition, adulterating foods leads to misrepresentation and may compromise food safety. High-value products, such as meat, are often susceptible to food fraud. Meat and meat products represent a vital component of the human diet [1]. Meat is a source of high biological value proteins, lipids, B vitamins (especially vitamin B12), and minerals such as iron and zinc. These macro- and micronutrients are essential for growth and body functions [2,3,4].

Meat has not often been associated with adulteration because it was marketed as fresh and domestically prepared [5,6]. Recently, there has been a shift in meat consumption patterns, with an increased consumption of processed and ready-to-eat convenience products [6,7]. These products include mince, patties, sausages, meatballs, and pâtés. When these products are created, there is a subsequent loss of morphological characteristics of the meat [8,9], making it increasingly difficult to differentiate between different muscle types and species. This affords people the opportunity to fraudulently replace or substitute meat of premium quality with inferior species or muscle types [5]. Consumers are adversely affected by these types of fraudulent activities because they can potentially lead to loss of income, consumption of meat prohibited in certain religions, unknowingly being exposed to food allergens, and general food safety concerns [10].

**Figure 1:**
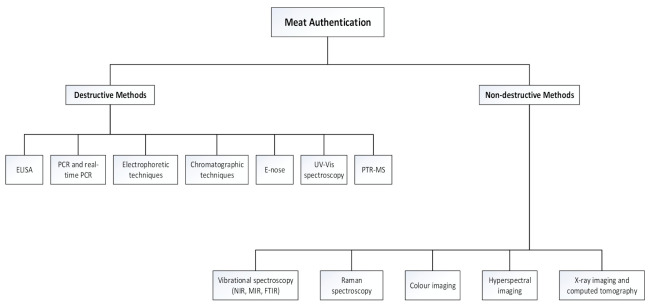
Schematic of the destructive and non-destructive methods used for meat authentication.

The meat production system is therefore challenged by these fraudulent activities and it is of vital importance that meat products are intensively controlled, monitored, and inspected during their processing, storage, and distribution [11]. Thus, in the fight to combat meat fraud, various analytical methods have been proposed and subsequently evaluated [12,13] (Figure 1). Some of the aforementioned methods include immunological detection and DNA-based techniques, e.g., enzyme-linked immunosorbent assay (ELISA) [7,14,15,16,17] and the now-infamous, polymerase chain reaction (PCR and real-time PCR) [18,19,20,21,22,23,24,25]. Electrophoretic and chromatographic methods [26,27,28,29,30,31] have also shown potential for this purpose. These methods are traditionally used on-/at-/in-line in a production plant to inspect, analyse, and assess raw and processed meat products based on quality- and safety parameters (presence of chemical, physical or microbial hazards and illegal substitution) [11]. Although reliable, specific, and sensitive, these authentication techniques have a number of drawbacks as they are destructive, time-consuming, expensive, and require complex laboratory procedures performed by skilled personnel which can make them susceptible to subjectivity. This renders these conventional methods inappropriate for rapid analysis and early detection, particularly under the fast-paced production and processing environment of the modern meat industry.

The mentioned disadvantages of the conventional methods have resulted in the development of rapid, non-destructive, non-invasive, accurate, reliable, and reproducible analytical techniques for authentication and adulterant detection in processed meat products. Developing a rapid, non-destructive method which can be implemented on- or at-line in a food processing environment has become an area of major interest in recent times. One such advancement has seen researchers make use of spectral- and imaging acquisition techniques, combined with chemometrics to evaluate meat and meat products in a non-destructive manner [32,33]. Techniques such as near-infrared (NIR) spectroscopy [34,35], Fourier transform infrared spectroscopy [8,9,36], Raman spectroscopy [36], colour imaging [37], hyperspectral imaging [38,39] and X-ray imaging [40] have been investigated for their application of authentication and/or adulterant detection in processed meat products. This review addresses and describes these non-destructive spectroscopic and imaging techniques that have been utilised to determine fraud in processed meat products along with their limitations (Table 1).

## 2. Non-Destructive Spectroscopic and Imaging Methods

In order for the meat industry to perform on-line analyses of processed meat products, without jeopardising the product, the development of non-destructive methods is essential. The term non-destructive analysis can be explained as the surface testing of samples in absence of any intrusive techniques which could negatively affect the food’s characteristics and quality. The main advantage of non-destructive spectroscopic and imaging methods is that the chemical and/or physical data of foodstuffs can be measured, without altering the substance. Consequently, obtaining quantitative and qualitative data in a single analysis. The application of non-destructive methods is therefore the best approach for fast-paced food processing plants because each sample can be analysed in its entirety, either on-line or in-line, without interrupting the processing line. With non-destructive methods, the analysed samples can be used in the supply chain, thus resulting in no product losses. Because the meat industry lacks rapid, non-destructive, and non-invasive methods that can be implemented in-situ for the authentication of processed meat products, only a few selected non-destructive methods were reviewed in this manuscript, as these have the potential to meet the industry’s needs. Hence, the goal of this review is to give an overview of the current developments of non-destructive spectroscopic and imaging techniques for the authentication of processed meat products. The general information of the selected studies reviewed is given in Table 2.

## 3. Vibrational Spectroscopy

### 3.1. Near-Infrared- and Mid-Infrared Spectroscopy

Vibrational spectroscopy is the detection of molecular vibrations and rotations that occurs when solid, liquid, or gaseous samples absorb infrared (IR) light [41]. The IR region of the electromagnetic spectrum consists of three sub-regions; near-infrared (NIR, 780–2500 nm), mid-infrared (MIR, 2500–25,000 nm), and far-infrared (FIR, 25,000–200,000 nm) [42]. However, the commonly used spectroscopic techniques for food authentication mainly focusses on the NIR and MIR regions.

The MIR region consists of absorption bands corresponding to fundamental stretching, bending, and rotating vibrations of the molecules related to chemical functional groups (O–H, C–H, C=O, and N–H) [42,43], whilst absorption bands in the NIR region result from complex overtones and combinations of fundamental vibrations [44,45,46,47,48]. Upon irradiation with MIR and NIR frequencies, vibrational energy changes occur in these molecular bonds [45,46,47,49]. Energy is absorbed by these functional groups when the light energy is equivalent to the frequency of the molecular bond.

Conventionally, IR instruments consist of the following parts: a light source, wavelength selection grating system, a detector, and a signal processor/amplifier, all of which are connected to a computer to study the output spectra [48,49,50]. Due to the robustness and simplicity of the instrumentation, MIR- and NIR spectroscopy have been used in food research for decades [50,51]. Although both methods are widely used, they have their own strengths and weaknesses, and these attributes make them better for certain applications. The main advantages of these techniques are that they require no sample preparation and are non-destructive [50]. Another advantage of NIR spectroscopy is the ability to make reflectance measurements in addition to the absorption and transmittance measurements typically used for MIR spectroscopy. In terms of sampling, some NIR instruments allow for the rotation of a sample while scanning. This allows for a larger volume/area to be scanned and therefore more representative sampling when compared to MIR. NIR spectroscopy is therefore a viable option for the measurement of inhomogeneous samples [49,52]. On the other hand, MIR spectroscopy has been shown to work best when using smaller volumes and homogeneous samples, making the detection of trace materials more difficult.

As mentioned, MIR spectroscopy measures fundamental vibrational bands related to the functional groups in the sample [42,43]. These fundamental bands result in spectra which are ideal for determining sample composition and identifying samples based on their unique MIR fingerprint. In contrast, NIR spectroscopy results in more complex spectra. The NIR region is characterised by spectral bands which are broad, extensive, and overlapping. These characteristics make it challenging to determine discrete chemical species [53]. Another difficulty of this technique is that the analytical information obtained may be influenced by a multitude of chemical, physical, or structural variables [54]. Many compounds absorb energy across the entire NIR spectral region and the small discrepancies in the spectra may very well be caused by inherent differences between samples, making a distinction with the naked eye nearly impossible. To mitigate this, multivariate data analysis techniques have been implemented which extracts the analytical data, buried in the NIR spectra. Chemometrics is thus essential to decompose the spectral data so that interpretation can be conducted accurately.

MIR spectroscopy was used to distinguish pure minced beef from that adulterated with offal (kidney, liver) [55]. Partial least squares regression (PLSR), canonical variate analysis (CVA), and soft independent modelling of class analogy (SIMCA) were investigated for the rapid identification and quantification of beef, kidney, and liver. The results showed that the predictive models of the combined partial least squares (PLS)/CVA technique have the potential to effectively distinguish between beef and offal types [55]. A SIMCA model was developed to discriminate between unadulterated and adulterated samples, achieving a classification accuracy of 92%. The SIMCA model was also challenged by investigating the effect of different adulteration quantities (10–100% *w*/*w*) on the model’s predictive power. The model achieved a 100% classification accuracy for the pure and adulterated samples, thus suggesting a detection limit for these adulterants of at least 10% (*w*/*w*). Although the authors could detect and classify these adulterants at a concentration limit of 10% (*w*/*w*), the adulteration level was still fairly high. Because adulterations would normally occur at lower adulteration quantities, it is advised that future research should focus on the application of this technique to samples with lower adulteration levels. Finally, the amount of added kidney and liver was quantified using PLS regression models. These models achieved standard error of prediction (SEP) values of 4.8 and 4.0% (*w*/*w*), respectively, for the kidney and liver calibrations [55]. Although Al-Jowder, et al. [55] presented a well-designed study, with detailed background and methodologies, the study lacked a few key aspects. Future studies should clearly state the number of samples investigated and how the data was split into calibration and independent validation sets. All of this can be detrimental to a study as an inadequate sample set size can result in models that are overfitted. Therefore, it is difficult to evaluate the true performance of the models and the reported accuracies must be treated with caution.

Morsy and Sun [34] evaluated the potential of NIR spectroscopy as a rapid and non-destructive tool for the detection and quantification of different adulterants in minced beef. PLS regression models were developed using cross-validation (leave-one-out) and verified with different independent data sets [34]. The models performed well, yielding prediction *R*^2^ values of 0.96, 0.94, and 0.95 with SEPs of 5.39%, 5.12%, and 2.08% (*w*/*w*) for minced beef adulterated with pork (n = 144, 10–90% *w*/*w*), fat trimming (n = 112, 10–80% *w*/*w*) and offal (n = 80, 2.5–30% *w*/*w*), respectively. Linear discriminant analysis (LDA), partial least squares discriminant analysis (PLS–DA), and non-linear regression models were developed and applied for the classification of minced beef into two classes (unadulterated or adulterated). The models performed excellently and yielded classification accuracies of 100%. The design and structure of the experiments answer the questions asked and the experiments are well documented, making them easy to repeat. A suitable number of samples and adulteration concentrations (including low levels) were used and split into calibration and validation sets. Therefore, valid conclusions could be drawn about the model’s true performance. While the calibrations developed are specific for minced beef and the mentioned adulterant types, the overall principles using NIR spectroscopy would be applicable for adulterant detection and quantification in other minced meat products.

Rady and Adedeji [35] investigated the use of visible-near-infrared (Vis–NIR) spectroscopy to evaluate plant and animal proteins as potential adulterants in minced beef and pork. Multiple chemometric techniques were investigated for adulterant classification, prediction, and wavelength selection. The samples were divided into consecutive class levels [35] and the data were split into training (75%) and testing (25%) sets. In the first classification level, the samples were differentiated as pure or adulterated. The pure samples included beef (n = 37), pork (n = 23), chicken (n = 37), texturized vegetable protein (TVP, n = 31) and wheat gluten (WG, n = 24). In contrast, the adulterated samples (1–50% adulteration level at increments of 2%) were beef separately mixed with pork (n = 210), TVP (n = 315), chicken (n = 315), and WG (n = 315); pork mixed with TVP (n = 315); and beef mixed with pork and TVP (n = 75). The classification models could classify the pure (100%) and adulterated samples (96%) with high accuracies. In the second classification level, the adulterated samples were classified into adulterant type. The identification of the individual adulterants was less successful with classification rates of 69–100% [35]. In the last level, the adulterants were quantified using PLS regression models. The prediction models showed relatively good performance, yielding *R*^2^ values of 0.78–0.86 and ratio of performance to deviation (RPD) values of 1.19–1.98, which is acceptable considering the large number of adulteration levels investigated. Therefore, Rady and Adedeji [35] concluded from their study that Vis–NIR spectroscopy has the potential to rapidly and accurately authenticate minced meat. This study simulated the possible adulteration conditions faced in the meat industry by incorporating various adulterants and many adulterant levels, particularly low levels. This makes the study applicable globally. The study is well-designed and the presented results look promising. Unfortunately, the authors failed to mention which classification methods were used to obtain the reported results, making it difficult for the reader to evaluate and replicate the study. Additionally, the authors advised that future research should increase the number of samples and extend the sources, for improved model performance, and to generate more robust and representative results.

### 3.2. Fourier Transform Spectroscopy

Fourier transform spectroscopy (FTS) is a vibrational spectroscopic technique that requires a Fourier transform (FT) algorithm to turn raw data into an optical spectrum [56]. The FT systems are spectrophotometers that utilises interferometers to modulate the electromagnetic signal and a computer to obtain the spectra. The spectral data, obtained from the interferometer, is pre-processed with the FT algorithm [57] and the instrument is therefore referred to as an FT instrument. In these instruments, all the wavelengths interact with the detector simultaneously and an FT is applied to the data to obtain a typical spectrum [43,58]. The light beams are split and subsequently recombined in the interferometer. This is accomplished by making use of mirrors. This technique is rapid due to the simultaneous measurement of wavelengths and it offers an improved signal-to-noise ratio.

FT can be applied to various spectroscopic techniques including optical spectroscopy, infrared spectroscopy (FTIR, FT–NIR), Raman spectroscopy, nuclear magnetic resonance (NMR), magnetic resonance spectroscopic imaging (MRSI), mass spectrometry, and electron spin resonance spectroscopy [56]. However, this method is most often applied in the context of infrared spectroscopy.

FTS has been used with success for the detection and quantification of adulterants in meat and minced meat products [8,9,36]. Meza-Márquez, et al. [8] studied the predictability of minced beef adulteration with various contaminants (horse meat, fat beef trimmings, and textured soy protein) using mid-Fourier transform infrared (MID–FTIR) spectroscopy and chemometrics. The authors prepared various mixtures containing minced beef and contaminants. Principal component analysis (PCA) and PLS regression models were developed for each contaminant mixture, to detect and quantify the contaminant within the minced beef. From the results, it is clear that the chemical composition of the samples had an influence on the resulting spectra, representing internal chemical characteristics of the different mixtures. Good correlations between the absorbance spectra and the percentage of adulteration were obtained. Additionally, SIMCA models were developed to discriminate between adulterated and unadulterated samples. The models were able to differentiate between the two classes and obtained a classification accuracy of 100% [8].

Schmutzler, et al. [59] developed and compared three different setups based upon FT–NIR to detect the adulteration of veal sausage with pork meat. These methods included a benchtop device (800–2500 nm) fit for laboratory use, a fibre optic probe (800–2500 nm) suitable for industry in- and on-line application, and a handheld spectrophotometer (1596–2396 nm) ready for on-site analysis. Veal sausages were adulterated with pork and pork fat at adulteration levels of 10–50% (*w*/*w*) at increments of 10%. Measurements were taken directly, through the polymer packaging and through quartz cuvettes. Chemometric models were developed for each adulteration percentage and FT–NIR device setup. Support vector machines (SVM) calibration models were developed, with the PCA scores as input data, and validated [59]. The authors reported that the laboratory- and industrial setup were able to detect meat and fat contaminants at a limit of detection of 10%. Both setups, with measurements performed through quartz cuvettes, obtained a classification accuracy of 100%. Alternatively, scanning the samples directly through the polymer packaging, with the fibre optic probe, resulted in a reduced classification accuracy of 91.7%. The handheld device successfully discriminated between unadulterated and adulterated (10% *w*/*w* adulteration threshold) samples. Contrary to the results obtained using the quartz cuvettes, a decrease in performance accuracy was observed for measurements obtained whilst scanning through the polymer packaging. Due to the overlap of the various PCA clusters and a loss in signal intensity, the SVM classification accuracy decreased to 83.3%. The handheld device was less successful when detecting low levels of fat adulteration (10% *w*/*w*). This resulted in classification accuracies of 83.3% for the quartz cuvette measurements and 75% for the measurements performed through the polymer packaging. Additionally, when the adulteration levels between 20% and 40% (*w*/*w*) were used, the classification accuracy improved to 100%. This represents a well-designed study as it incorporates all aspects of the meat industry, from the development of the method at the laboratory scale to the use of NIR spectroscopy for on-site authentication and at point of sale through the packaging.

Alamprese, et al. [9] recorded the FT–NIR spectra of 198 samples (n = 154 adulterated, n = 22 beef, n = 22 turkey) in either their raw, frozen-thawed, or cooked state. Subsequently, different multivariate regression and class-modelling strategies were applied in order to identify and quantify turkey meat adulteration within minced beef. PLS regression with cross-validation (leave-one-out) was used to develop a model to predict the percentage of turkey meat in the minced beef. These models performed well and the reported errors are root mean square error of calibration (RMSEC) = 7.62–9.68%, root mean square error of cross-validation (RMSECV) = 9.64–10.84% and root mean square error of prediction (RMSEP) = 9.60–10.84% with *R*^2^*_cal_* = 0.89–0.93, *R*^2^*_cv_* = 0.87–0.89 and prediction *R*^2^ = 0.88–0.92% [9]. A valid conclusion can be drawn regarding the models’ predictive power as an independent validation set was used to test the performance of the cross-validated models. Furthermore, Alamprese, et al. [9] wanted to classify the meat samples into adulterated and unadulterated samples. The authors set a threshold of 20% according to the PLS regression results and investigated the ability of PLS–DA to differentiate between the two pre-defined classes. The performance measures for the different PLS–DA models (calibration, cross-validation, and independent validation) were similar and showed prediction values of sensitivity and specificity higher than 0.84 and 0.76, respectively. The PLS–DA models were thus able to accurately distinguish between low (<20%) and high (≥20%) levels of adulteration. Although the authors only reported the performance statistics, which is not incorrect, future research is advised to also include the percentage accuracies and errors because these are easier to understand and interpret. Alamprese, et al. [9] concluded from their study that it was possible to identify and quantify turkey meat adulteration in fresh, frozen-thawed, and cooked minced beef samples using FT–NIR spectroscopy.

### 3.3. Raman Spectroscopy

Raman spectroscopy is a scattering technique which is based on the Raman effect [60]. The Raman effect is defined as the scattering of light from molecules, which results in shifted energy frequencies [42,60]. The resulting frequency shift contains information about the vibrational modes involved and is used to construct a Raman spectrum [61,62]. Raman signals result from the inelastic scattering and collision of light and molecules from a sample. In Raman spectroscopy illumination of the sample is attained by making use of a monochromatic laser beam [60]. Electromagnetic radiation is collected from the illuminated spot and is then sent through a monochromator. The scattered radiation is filtered out by one of a variety of filters, such as an edge pass filter, notch filter, or a bandpass filter, and the remaining light is dispersed onto a detector.

Raman spectroscopy is a commonly used technique to provide a unique structural fingerprint whereby molecules and compounds may be identified in samples. These fingerprints also provide the basis for structural, qualitative, and quantitative analyses [60,63]. The frequency of the scattered radiation is measured to achieve qualitative analysis, while the intensity of the scattered radiation is measured for quantitative analysis [64,65]. Raman spectroscopy has at least 25 variations [66], developed with the purpose to enhance the sensitivity (surface-enhanced/tip-enhanced Raman), to improve the spatial resolution (Raman microscopy), or to acquire very specific information (resonance Raman). The combination of Raman spectroscopy with chemometrics enables researchers to determine food adulteration rapidly [67,68] thereby, demonstrating the potential of this technique as an authentication method.

Boyacı, et al. [69] studied the predictability of meat adulteration using Raman spectroscopy. This research was conducted on 49 samples (18 horse, 31 beef) with the samples being split into calibration (n = 39) and validation (n = 10) sets. The calibration data was used to develop a PCA model to distinguish between the pure beef (n = 25) and horse (n = 14) samples. The scores of PC1 (96.32%) and PC2 (3.20%) were used to distinguish between the horse and beef samples. The validation set (n = 6 beef and n = 4 horse) was then used to assess the performance of the model, resulting in the successful clustering of all the meat samples according to their origin. The PCA model was also used to differentiate between the different horsemeat adulteration concentrations (0%, 25%, 50%, 75%, and 100% *w*/*w*) within the beef [69]. It is important to highlight, that Boyacı, et al. [69] tested the performance of their PCA calibration model with an independent validation set. Although the performance of the developed model system was reported as being good enough for the determination of meat samples, PCA is mainly used for exploratory purposes and not classification. Classification techniques such as LDA, SIMCA, and PLS–DA are routinely used, and it would have been interesting to see the performance of these correct techniques.

Raman spectroscopy has also been investigated to detect and quantify adulterations in beef burgers [70]. The authors prepared 81 beef burgers (36 authentic, 45 adulterated) containing authentic beef and adulterants (kidney, liver, heart, lung). Zhao, et al. [70] developed and independently evaluated their models by splitting the data set into calibration (50%) and validation (50%) sets. Using PLS–DA and SIMCA, chemometric models were developed to identify offal-adulterated and authentic beef burgers. The various PLS–DA models for the calibration set were effective when classifying the authentic (89–100%) and adulterated (90–100%) samples. When investigating the performance using the validation step, the identification of authentic samples remained in the 94–100% range, however, the classification of the adulterated samples suffered performance losses, with a classification accuracy between 81–90%. Unfortunately, the authors gave no final inference on this phenomenon and further investigation should be conducted. The results of the SIMCA method indicated that the models were able to authenticate the pure beef burgers (sensitivity value of 1) but could not identify the offal-adulterated beef burgers accurately (specificity value <1), thus resulting in a few false-positive classifications. The classification accuracy of the SIMCA models can be improved by using the PLS scores and applying the test samples in parallel to the PLS–DA models. This approach allows for the samples to be identified with greater confidence as both model types are used. A PLS regression method was also developed to quantify the total offal and added fat in the samples [70]. Unfortunately, the regression method was not able to predict the offal content. This was expected due to the inability to identify a unique spectral signal for offal due to the inherent complexity of the substrate and the meat itself. The performance of the PLS regression models to quantitatively predict added fat was also not satisfactory for use as an analytical method, hence this method may only be suitable for screening purposes. Zhao, et al. [70] therefore indicated that using Raman spectroscopy to investigate a complex meat formulation resulted in some misclassification and quantification errors. These authors advised that future research requires extension and should include the use of multi-spectroscopic methods (e.g., NIR, MIR) combined with Raman spectroscopy [70]. This suggests that this strategy has the potential to minimise sample misclassification because these spectra contain complementary information and could therefore improve classification and quantification outcomes in complex adulteration problems.

## 4. Colour Imaging

**Figure 2:**
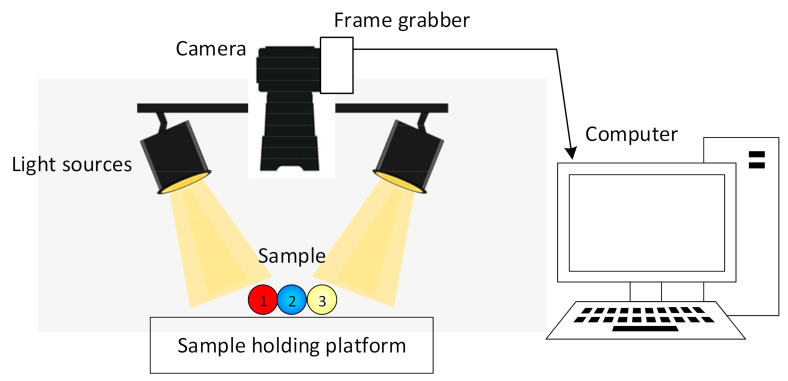
Schematic of a typical colour imaging system adapted from Vithu and Moses [73].

Colour imaging permits the determination of the general colour and visual appearance of a sample [71]. Colour images can be represented in two ways, namely, through the three primary colours red, blue, and green (RBG), or through the main factors influencing colour sensation, namely, hue, saturation, and intensity (HSI system) [72]. An image acquisition system comprises a platform upon which the sample is placed, a digital camera, image capture board (frame grabber or digitiser), light source, computer hardware, and finally image analyses software (Figure 2) [73]. To acquire an image, this technique relies upon the incident light in the visible range (400–750 nm) interacting with a partially reflective surface of the sample being investigated. The camera lens collects the scattered photons and converts them to electrical signals either by a vacuum tube or by a charged coupled device (CCD) [74]. Electrical signals are then saved to a hard disc to allow for further image display and analysis.

Image processing and image analysis are two key features for the application of colour imaging [73]. Image pre-processing is not meant for extracting information from the acquired digital image but rather for the purpose of enhancing the image quality or for the removal of irrelevant sources of variation (i.e., noise, blurring) [73,75]. The colour features of an object are then extracted by examining each pixel within the object boundaries, using appropriate image processing algorithms [72,76]. These features are then used as input to statistical discriminant classifiers to differentiate foodstuffs. Colour imaging has the advantage of analysing the entire surface of foodstuffs pixel-by-pixel and quantifying the surface characteristics [72,76]. Therefore, colour imaging has extensively been utilised in the food industry as a rapid, economic, consistent, accurate, and non-invasive technique [75,77].

de Sousa Fernandes, et al. [78] investigated the development of a method for the quantification and identification of fat content adulteration in chicken burgers using digital images and chemometric tools. In this work, 74 chicken burger samples with a fat content of 14.27–47.55% (*w*/*w*) were prepared and studied because Argentinean legislation states that the total fat in burgers should not exceed 20% (*w*/*w*) [79]. Chemometric models (PLS, PLS–DA, and successive projections algorithm (SPA–LDA)) were developed using different colour histograms in the Grayscale, RGB, HSI channels and their combinations as analytical information [78]. The authors found that the best results for quantification and identification were achieved by the chemometric models containing HSI histograms. For fat quantification, the PLS/HSI model achieved the best result with a prediction *R*^2^ value of 0.95, RMSEP of 2.01% *w*/*w*, a relative error of prediction (REP) of 7.26% *w*/*w*, and RPD of 4.47. While for fat identification the SPA–LDA/Grayscale + HSI histogram model achieved satisfactory results and could differentiate between the adulterated and non-adulterated burgers with classification accuracies of 92% (training) and 95.8% (test set), respectively. This was a well-designed study because the authors based their research on Argentinean legislation and incorporated retail samples to evaluate their models, therefore making this study applicable to the meat industry. The authors presented a well-explained methodology, making this research easy to follow, understand, and replicate. It is also very easy to assess the true predictive power of their chemometric models because the authors presented calibration, cross-validation, and independent validation data for each of the developed models. Therefore de Sousa Fernandes, et al. [78] successfully demonstrated the potential of a digital image-based method to monitor fat content adulteration in chicken burgers. Although this method achieved excellent results, future research should increase the number of samples and could incorporate a variety of meat types (e.g., beef, pork, mutton), for improved model performance and to generate more robust and representative results.

The performance of colour imaging to differentiate ground meat samples from beef, pork, and chicken and to quantify the adulteration of ground chicken with pork and beef was evaluated by Nolasco-Perez, et al. [37]. This work consisted of 420 samples from two adulterated batches (210 adulterated with pork, 210 adulterated with beef) and each adulterated batch was separated into calibration (n = 135) and validation (n = 75) sets. Colour images were used to develop classification and regression models using LDA and PLSR. The LDA results showed the potential of RGB-imaging to discriminate between the three classes (beef, chicken, pork), by obtaining an overall accuracy of 100%, with a sensitivity and specificity of 1.00 [37]. Thus, confirming the suitability of this method for the rapid and accurate classification of different species. PLS regression models were also developed to predict the concentration of pork (0–50% *w*/*w*) and beef (0–50% *w*/*w*) when added to ground chicken. The models performed well and could predict the level of pork and beef adulteration in the chicken samples with a high degree of accuracy. The model for pork adulteration had a prediction *R*^2^ value of 0.82, RPD of 2.38, and range error ratio (RER) of 9.84, indicating that model was good for predicting the concentration of pork [37]. For the chicken samples adulterated with beef, the model had a prediction *R*^2^ value of 0.86, with RPD and RER values of 2.66 and 10.99, respectively. This indicates that the model predicted the level of beef adulteration with excellent accuracy. Additionally, Nolasco-Perez, et al. [37] reduced the image features in order to improve and optimise the PLS regression models. The authors however did not report on which chemometric approach they used to select a few image features. Nevertheless, the study was well designed, thoroughly explained and all the necessary calibration, cross-validation, and prediction results were reported. Hence, a reasonable statement regarding the feasibility of the technique could be made. Although the purpose of the study was to develop a method for on-line inspection, the research was conducted on laboratory scale. It would have been interesting to see whether this technique would be adequate during on-line meat processing for species identification and adulterate detection/quantification.

## 5. Hyperspectral Imaging

Conventional NIR spectroscopy has one shortcoming—it only provides spectral information about an entire sample in one spectrum. This means that no spatial information can be retrieved from conventional NIR spectroscopy. Hyperspectral imaging (HSI) on the other hand incorporates spatial and spectral information and thereby overcomes this shortcoming of conventional NIR spectroscopy [80]. A new technique, at the time, that combined digital imaging and spectroscopy was first used by Goetz, et al. [81]. Over time, this technology was adapted and eventually evolved to be suitable for use in laboratory experiments and it has been applied in a variety of applications. It is frequently used in the food, agricultural and pharmaceutical industries [51]. NIR–HSI is superior to that of conventional NIR spectroscopy as it provides also spatial information, thus allowing the visualisation of constituents within a measured sample [82,83].

Hyperspectral images consist of hypercubes, which are three-dimensional data cubes, which consist of numerous contiguous wavebands for every spatial position of a specific sample [51,84]. For every pixel represented in an image, a corresponding spectrum exists. Each individual spectrum can act similar to a chemical fingerprint, which allows for the identification of chemical constituents in a given sample [84,85]. Hyperspectral imaging can visualise the distribution of the various chemical components in a sample, a distinct advantage over conventional NIR spectroscopy. This allows for the analysis of heterogeneous samples and identification of the chemical components that constitute the sample [52,82,84].

**Figure 3:**
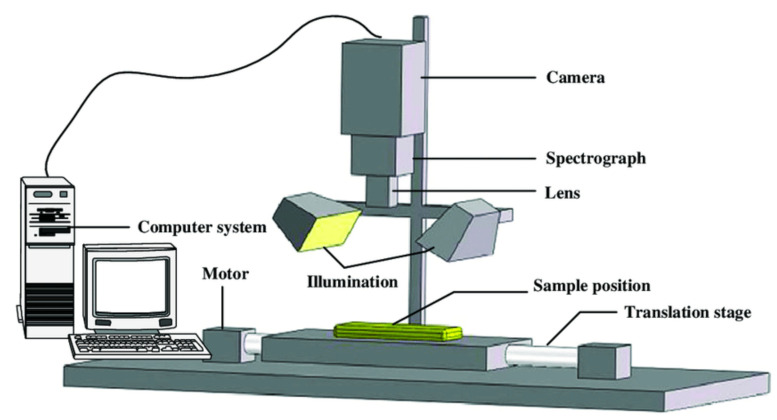
Schematic diagram illustrating the main components of a line-scan hyperspectral imaging system [87]. Reprinted with permission from ref. [87]. Copyright 2014 Elsevier.

**Figure 4:**
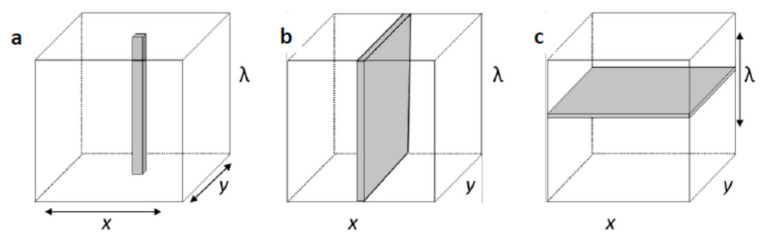
Three configurations of hyperspectral imaging systems. (**a**) Whiskbroom or point scan configuration—scans the sample in point form. (**b**) Push-broom or line-scan configuration—scans the sample in consecutive lines. (**c**) Staredown configuration—scans entire sample (within camera frame) one wavelength at a time [88]. Reprinted with permission from ref. [88]. Copyright 2013 Elsevier.

A typical NIR–HSI system is composed of a camera, a spectrograph, and detector, an illuminator, and a translation stage all coupled to a computer (Figure 3) [84,86]. There are three imaging configurations or means of generating a hyperspectral image from a sample, i.e., whiskbroom imaging (point scan), push-broom imaging (line scan), and staredown imaging (wavelength scan) (Figure 4) [84].

The whiskbroom configuration (Figure 4a), allows for the acquisition of hypercubes by scanning single points of a sample (pixel-by-pixel), by either moving the object or the detector in the spatial direction. Although time-consuming, this technique yields high spatial resolution images and is, therefore, most suitable for microscopic imaging [84,88]. The push-broom configuration (Figure 4b) is the fastest method and one image acquisition can be performed within 20 s, depending on the field-of-view and magnification [85]. A two-dimensional detector is used to obtain the spatial and spectral data line-by-line and is therefore well-suited for at-line or on-line applications on conveyor belts [84,86,89]. Within the staredown configuration (Figure 4c), the spectrophotometer components and the sample remain stationary while an image of the entire field-of-view is acquired [83,84]. The HSI instruments collect a sequence of images, one wavelength band at a time. The collected images are used to construct a three-dimensional image, using the stack of images, known as a hypercube [84,86,88].

All biological materials either reflect, scatter, absorb, or emit electromagnetic energy. The pattern in which these materials interact with the light is distinctive for a specific wavelength. This forms the basis for HSI. This phenomenon can be ascribed to the differences in their chemical composition and inherent structure [32]. The characteristic pattern obtained from the interaction of light with a specific material is known as a spectrum/spectral signature/spectral fingerprint. The unique spectral signature can then be used for the identification, characterisation, and discrimination of sample types and classes [32]. Spectroscopic data often contain disturbances caused by scattering due to surface inhomogeneity [86]. Spectral pre-processing is performed to eliminate specific non-chemical biases from the acquired spectra before extracting the most relevant analytical information [51,54]. The data is then subjected to sophisticated chemometric techniques to extract the most meaningful information from the data matrix as well as highlight possible differences between the analysed samples [51].

There is a growing interest in this technique, attributable to its advantages above the alternative instrumental techniques. Due to increasing quality and safety concerns, it has become important to have the ability to trace specific molecules, chemicals, or constituents within samples. NIR–HSI can record spectra of samples instantly (at-line) and continuously (on-line) to predict the chemical, and the physical, parameters of the sample from a single measurement [51,54]. These features allow for the rapid, non-destructive analysis of samples, which could allow for superior quality control and process monitoring in a factory environment, specifically for on-line and at-line applications.

Kamruzzaman, et al. [33] investigated the possibility to differentiate between pork (n = 75), beef (n = 75) and lamb (n = 75) using NIR–HSI. In this study, the data set consisted of 225 meat samples which were split into calibration (n = 150) and validation (n = 75) sets using the Kennard–Stone algorithm. The authors used the second derivation of Savitzky–Golay (SD–SG) to reduce the dimensionality of the data and to identify six optimal wavelengths (957, 1071, 1121, 1144, 1368, and 1394 nm) that were selected for the basis of discrimination. The calibration and validation in the PLS–DA model yielded accuracies of 97.33% and 98.67%, respectively. Although the use of NIR–HSI looks promising, it should be remembered that this investigation was carried out in a laboratory (off-line) and therefore future research should be implemented at an industrial scale to investigate the feasibility of this technique for on-line applications. Because the conditions in a factory and laboratory differ, the models might have to be adapted or changed before on-line implementation. It is important to highlight that optimum wavelengths were selected, indicating that a multispectral imaging system could easily be installed over the conveyer belt with minimum modifications of the industrial set-up [33].

In 2013, this research was expanded by the same research group [89] who aimed to develop a non-destructive method that could detect and quantify the level of adulteration in processed meat products. In this later study, 40 samples of minced lamb were adulterated with pork (2–40% *w*/*w*). The authors performed multivariate data analysis, to predict the level of adulteration, in two ways—firstly, they used the full wavelength range (910–1700 nm) to develop a PLSR model, and secondly, they selected four important wavelengths and developed a multiple linear regression (MLR) model and evaluated the prediction ability of the PLSR model using these selected wavelengths. The PLSR calibration model yielded an *R*^2^*_cal_* = 0.99 and an RMSECV of 1.08%. After cross-validation (leave-one-out) the model yielded a *R*^2^*_cv_* = 0.99 and a slightly increased RMSECV = 1.37%. In the case of the MLR model, similar values were reported for calibration and cross-validation with a *R*^2^*_cal_* = 0.99, RMSEC = 1.25%, *R*^2^*_cv_* = 0.98, and a RMSECV = 1.45%. Additionally, Kamruzzaman, et al. [89] reported that similar prediction results were observed for the PLSR (full- and selected wavelengths) and MLR models. Finally, the MLR model was selected because it is simple and easy to interpret compared to the PLSR models. Although the values reported in this study are promising, it should be remembered that a small sample set was used and that no independent validation set was applied to the cross-validated models to test their performance. Because no independent validation set has been predicted with acceptable RMSEP values, it is difficult to assess the feasibility of NIR–HSI to predict pork adulteration in lamb meat.

In a similar study, Kamruzzaman, et al. [38] investigated the feasibility of a visible near-infrared (Vis–NIR) hyperspectral imaging system to detect horsemeat adulteration (2–50% *w*/*w*) in 38 minced beef samples. The authors selected four important wavelengths (515, 595, 650, and 880 nm) using the weighted regression coefficients obtained from the PLSR model. The calibration and cross-validation (n = 25 samples) of the PLSR models were performed with reported standard error of calibration (SEC) < 1.38% and standard error of cross-validation (SECV) < 2.66, with *R*^2^*_cal_* = 0.99 and *R*^2^*_cv_* = 0.99. Kamruzzaman, et al. [38] then evaluated the true performance of the PLSR models with an independent validation set (n = 13 samples). The level of horsemeat adulteration was successfully predicted with an *R*^2^ value of 0.98 and a SEP < 2.63%. The authors made use of an independent validation set for result verification. This supported their conclusion that a laboratory-scale HSI instrument can be used as a rapid, non-destructive technique to detect adulteration in minced meat. The design and structure of the experiments answers the questions asked and the experiments are well documented. For this technique to be considered for on-line applications, robust models with a large sample set are required to include all possible variations, therefore additional research with an increased sample size is needed to test the robustness of this methodology before this technique can be utilised in on-line applications.

Zheng, et al. [39] also studied the use of Vis–NIR hyperspectral imaging (400–1000 nm) for the detection of duck meat adulteration (0–100% *w*/*w* at 5% increments) in 63 minced lamb meat samples. Fourteen selected wavelengths, determined by SD–SG, were used for the PLSR model, achieving excellent prediction results with an *R*^2^ value of 0.99 and an RMSEP of 2.51%. Zheng, et al. [39] advised that future research should consider different breeds of lamb and duck when sampling to incorporate biological variability. In this study, an on-line system was used, demonstrating the potential implementation of this method into production practice.

Minced pork adulterated with minced pork jowl meat was investigated, using Vis–NIR hyperspectral imaging, to identify this type of meat adulteration [90]. Minced pork samples (n = 176) were adulterated with pork jowl meat in the range of 0–100% (*w*/*w*) at 10% increments. The spectral data were used to develop a PLSR calibration model (n = 132 samples) to predict the adulteration level of an independent validation set (n = 44 samples). Full PLS regression (400–1000 nm; 284 spectral bands) resulted in an *R*^2^*_p_* of 0.96, RMSEP of 7.04%, and RPD of 4.54. Three waveband selection methods were further explored, using principal component (PC) loadings, two-dimensional correlation spectroscopy (2D-COS), and regression coefficients (RC) [90]. PC loadings, 2D-COS, and RC were each applied to PLS regression based on the individually selected wavelengths related to the adulteration identification. The RC–PLSR approach, with 10 selected wavebands, performed the best with an *R*^2^*_p_* of 0.91, RMSEP of 13.93%, and RPD of 2.30. Unfortunately, PC loadings and 2D-COS performed poorly due to the lack of suitable spectral information and this can be attributed to how the wavelengths were selected. These two approaches only use the X-variables (spectra) when selecting wavelengths, whereas RC selects the wavelengths based on the PLSR model which utilises both X- and Y-variables (adulteration levels) in the calculation. The RC approach optimises the relationship between the spectral data and adulteration levels, thus resulting in wavelengths containing sufficient information. The model also achieved a limit of detection (LOD) of 6.50%. While the number of wavebands was reduced to 4% of the original set, the authors failed to link these key wavebands to any chemical components in the pork or jowl meat. Therefore, a comment on the chemical components that could be responsible for driving the prediction would have been interesting.

The aforementioned studies demonstrate the potential of HSI, paired with multivariate data analysis and image processing, as a rapid, non-destructive, and objective method for the authentication of minced meat products [33,39,40,90,91]. These studies also highlighted the recent developments in spectral imaging research by investigating strategies for optimal waveband selection. These approaches aim to improve the accuracy and the simplicity of applications by the removal of redundant spectral information. The preliminary hyperspectral imaging research reviewed indicates the starting point for the development of industry-scale multispectral imaging systems. It is possible to produce a multispectral imaging device that is a cheap, rapid, and reliable alternative to traditional analytical methods by optimising wavelength selection for a specific application [39,40,91].

## 6. X-Ray Imaging and Computed Tomography

X-rays are high-energy electromagnetic radiation (0.01–10 nm) that can pass through matter. The principle of X-ray imaging as an inspection technique is based on density differences between the product (light grey) and the contaminant (dark grey) [91]. Because an X-ray photon penetrates and exits a food product, it reaches a sensor which converts the energy signal into an image. The image is then used to directly identify internal defects or foreign contaminants and internal structural changes [91]. X-rays can either be classified as soft X-rays (wavelengths ranging from 0.1–10 nm with corresponding energies of 0.1–10 keV) or hard X-rays (wavelengths ranging from 0.01–0.1 nm with corresponding energies of 10–120 keV). Due to their penetrating abilities, hard X-rays tend to pollute food, therefore soft X-rays are more suitable for use in food inspection [91,92]. Soft X-ray imaging is rapid and to produce an X-ray radiograph only takes 3–5 s [93]. Radiographs (projection/shadow images), project a 3-D object on a 2-D detector plane, thus resulting in loss of depth information [94]. This problem was overcome through the development of computed tomography (CT) which is an X-ray imaging technique that allows for the visualisation of 3-D images. This is accomplished by making use of computer algorithms (Radon transform) that can reconstruct a 3-D volume/image, utilising multiple projections from an array of different angles or directions [95]. The image obtained makes an in-depth analysis of the internal structure of an object possible. Images produced by CT scans are superior in quality when compared to traditional X-ray imaging systems, but they have the disadvantage of being expensive (extensive volumes of lead required for safety purposes) and have lengthy image acquisition and data processing times [96].

X-ray CT is based on image contrasts produced by differences in the X-ray attenuation and are attributable to density and compositional differences within the sample [97,98]. The contrast, created by the difference in X-ray attenuation, is then used to differentiate low- and high-density regions within the sample [99]. A typical X-ray imaging setup consists of an X-ray source (illuminator), a sample manipulator, and a detector [92]. During scanning the sample rotates on a translation stage while the X-ray source and the detector remain fixed [94,100]. The X-rays pass through the sample in a number of different directions as well as different pathways, creating an image which illustrates the variation in density at numerous points in a two-dimensional slice [101]. As the sample rotates, the X-ray CT acquires a series of two-dimensional radiographs or projection images [102]. The total angle of rotation is dependent upon two factors, namely; the geometry of the beam and the sample. In the case of a parallel beam the angle is typically at 180°, while the use of a cone-beam results in a 360° angle [100].

The series of 2-D projections/slices can be reconstructed into a three-dimensional image and can be presented as the whole sample, or as virtual samples at differing depths and in different directions [102]. Therefore, X-ray CT enables both 2-D and 3-D visualisation of the sample’s internal structure and quantitative characterisation of the data volumes [101]. The quantitative results are obtained using dedicated software packages to extract valuable information from the X-ray images [94]. Quantitative results that can be obtained from the 3-D analysis include the volume of the sample, density, pore size and distribution, object surface to volume ratio, morphology (shape, sphericity, and roundness), and surface texture among other characteristics [94,98]. The quantitative parameters are then used to aid in the discrimination of different classes.

X-ray micro-CT (65 kV, 170 µA) was used to study the non-invasive detection of mechanically separated meat (MSM) in processed meat products, based on the detection of bone fragments as indicators [40]. A cooked meat sausage containing 50% of MSM was used for this study. Details of the sample’s inner structure could be visualised in the X-ray µCT images according to the absorption properties, and thus the parameters of the individual bone fragments were quantified in terms of volume, length, circumference, diameter, and surface area. The bone tissue was easily detected due to its high density, as confirmed by the image analysis and histochemical method, making it a more absorbing material compared to soft tissue [40]. Therefore, the authors verified this method as being suitable and non-destructive for the analysis of bone fragments in meat products with the possibility to detect MSM. This study was well explained and the results look promising, but a sample set of one is inadequate to make any conclusions regarding the performance of this method.

X-ray imaging has the advantage to non-destructively detect hidden defects or contaminants by visualising the interior features of a sample. However, X-ray µCT also has a few limitations which might explain the lack of popularity of this method for meat applications. The limitations include operator dependency, cost, and time constraints as the method requires lengthy image analysis procedures [98]. The safety and amount of lead required could also pose a challenge for the industrial implementation of this technique.

## 7. General Discussion and Recommendations

### 7.1. Experimental Design

The mentioned studies were well-designed and the structure of the experiments answered the questions asked. Most of the research reviewed gave sufficient detail on the methods used and the implementation thereof. The experiments were well documented and would thus be easy to repeat. Although these studies highlight the potential of non-destructive spectroscopic and imaging methods for the authentication of processed meat products, one critical point to consider is the lack of a sufficient number of sample and sample types to challenge the models developed. The majority of current research only investigates minced meat products and therefore lacks the authentication of processed meat products such as patties, sausages (fresh and fermented), meatballs, and pâtés. Therefore, additional research with an increased number of samples, sample types, and samples consisting of complex meat matrices is needed before these techniques can be utilised in on-line applications. Even though the reviewed studies highlight the potential of the non-destructive methods for the authentication of processed meat products, one should remember these were performed on a laboratory scale and consequently considered as proof-of-concept studies. Therefore, these studies serve as a baseline for future research.

### 7.2. Multivariate Data Analysis

It is becoming increasingly evident that researchers make use of spectral- and imaging acquisition techniques to evaluate meat in a non-destructive manner. The development of these techniques requires the use of multivariate data analysis approaches that consists of two phases, spectral pre-processing and spectral processing [107].

The articles reviewed all investigated the performance of various pre-processing techniques used to eliminate specific non-chemical biases from the data often containing disturbances due to scattering [54,86]. Therefore, pre-processing is performed to prepare and improve the data, in order to develop accurate, robust, and reliable models. Although a wide variety of pre-processing techniques are available the studies reviewed mostly used multiplicative scatter correction (MSC), standard normal variate (SNV), de-trending (DT), and derivatives, including Savitzky–Golay or a combination of these techniques. On a positive note, it is reassuring that the researchers kept the pre-processing to a minimum, therefore avoiding over pre-processing of the data and removing of valuable information.

The spectral processing step includes data mining and the construction of classifiers. Data mining, also referred to as data exploration, is used to familiarise oneself with the data. The process is used to extract information from the data matrix, detect clusters, identify discriminant spectral features and outliers. Principal component analysis (PCA) is a powerful and frequently used technique and was correctly implemented by all the authors. After data exploration and the removal of outliers, classification and regression models were built using several multivariate approaches. Because there is no general consensus of the best technique, researchers would often investigate various supervised algorithms. The techniques most frequently used in these studies were SIMCA, LDA or PLS–DA, SVM, PLSR, and MLR. All the herein reviewed studies, with the exception of one study, correctly applied the data analysis algorithms. Although the authors reported good accuracies for their models, future studies should consider other classification techniques, e.g., K-nearest neighbour (KNN), artificial neural networks (ANN), backpropagation neural network (BPNN), random forest (RF), or decision tree.

A crucial step during model development is the assessment of the model’s performance using an independent validation set. Therefore, the data needs to be split into calibration and validation sets using appropriate methods, e.g., the Kennard–Stone algorithm. It is also important to include an internal cross-validation step (leave-one-out cross-validation was often used in these studies), to ensure that the calibration model is not over- or under-fitted.

A few of the studies also employed waveband selection methods (PC loadings, 2D-COS, and RC) which were reported to have greatly improved the models’ prediction capabilities in meat fraud applications. The accuracy and simplicity of the applications were improved by selecting optimal wavebands containing the most useful information. The advantages of waveband reduction are that data acquisition and analysis times are reduced and systems using simpler and less expensive components can be developed. Hence, the development of such systems could potentially result in the implementation of spectral- and imaging acquisition techniques for the on-line authentication of processed meat products at an industrial scale.

## 8. In-Situ Applications

It is evident that there is an abundance of studies dedicated to the rapid, non-destructive and non-invasive authentication of meat products, however, the research is lacking in terms of published work where methods have been applied in-situ. Unfortunately, only a limited amount of research has been published on in-situ applications and this scenario needs to be investigated further.

The in-situ authentication of Iberian pig carcasses was investigated using a NIR spectroscopy handheld device [104,105,106]. According to Spanish legislation, Iberian pig products are categorised based on the animals feeding regime and labelled accordingly. In the study of Zamora-Rojas, et al. [104] Iberian pig carcasses (n = 279) from two slaughterhouses were scanned 2 h post-slaughter and classified based on the three feeding regimes, e.g., ‘Acorn’, ‘Recebo’, and ‘Feed’. The authors kept 94 samples out of the PLS–DA calibration (n = 185) for the independent validation of the developed model. Zamora-Rojas, et al. [104] reported successful classification accuracies for the ‘Acorn’ (93.9%) and ‘Feed’ (96.4%) carcasses with the ‘Recebo’ carcasses achieving lower accuracies of 60.6%. Although the accuracy for the ‘Recebo’ was low, the overall performance of the handheld instrument showed the potential of such a technique to be implemented on the slaughter line.

A more promising approach for the authentication of Iberian hams seems to be the classification of meat samples into ‘premium’ and ‘non-premium’ categories [105]. The authors used a portable NIR spectrophotometer to measure the qualitative categories of 495 Iberian pork carcasses (n = 265 premium grade, n = 230 non-premium grade) from 45 different slaughterhouses. Data analysis was conducted using PCA and three chemometric approaches—LDA, quadratic discriminant analysis (QDA), and non-parametric Bayes (NPB). The spectral data were used to develop calibration models (n = 295 samples) to predict the predefined categories of the independent validation sets (n = 200 samples). The authors mentioned that an internal validation step was performed on the calibration sets, but it is unclear as to which cross-validation method was used during calibration. The results for the individual models were presented as confusion matrices, and although this was not incorrect, presenting the data as classification accuracies and errors would have made the data easier to read, understand and interpret. The overall results for the LDA, QDA, and NPB approaches were almost identical and the reported error rates were 3.1–5.0% (calibration sets) and 1.5–2.5% (validation sets). Piotrowski, et al. [105] reported acceptable results and concluded that 98% of the independent samples were correctly classified. This study highlighted the potential of a portable NIR spectrophotometer, used in-situ at a slaughterhouse, for the authentication of Iberian pork carcasses.

Similar to the previous study, Pérez-Marín, et al. [106] explored four discriminant methods (LDA, QDA, Kernel Bayes, and logistic regression) to classify Iberian pig samples into two categories using a portable handheld NIR spectrophotometer. In this study, 495 carcasses from 45 producers were measured 2 h post-slaughter in-situ at the slaughter line. The data was split into a training- (n = 369 samples) and test set (n = 199). The training set along with the performance of internal cross-validation (leave-out-one-producer) was used to develop the various discriminant models. Thereafter the performance of each calibration model was assessed with a separate independent test set. The authors reported that the LDA model performed best when classifying the samples into ‘premium’ (acorn fed animals) and ‘non-premium’ (compound feed fed animals) categories. The calibration in the LDA model yielded an error rate of 7.9%, which improved to 6.5% after the prediction of the validation set. Pérez-Marín, et al. [106] concluded that the use of a portable NIR spectrophotometer opens the avenue for many new possibilities for the assessment of food integrity and authenticity issues. This study was well designed with the experiments and methods being well documented.

Based on these reviewed studies, it is evident that a portable NIR device can be used in-situ at a slaughterhouse for the authentication of meat products. While the calibrations developed are specific for Iberian pork products and thus only appropriate in Spain, the overall results using portable NIR technology would be applicable globally. Thus, these studies can be seen as a baseline, and therefore future research should investigate other meat samples and consider using additional portable devices, e.g., handheld FTIR spectrophotometer, handheld Raman spectrophotometer, RGB-imaging using smartphones, and a handheld HSI device. Although these portable handheld devices exist, they are still in the infancy stage. Therefore, the development of miniaturised and portable instruments needs to advance before they can be utilised for the on-line inspection of meat products. With the necessary instrument improvements, these devices might offer promising alternatives to the expensive, destructive, and time-consuming conventional methods used for meat authentication.

## 9. Challenges and Future Trends

Despite the rapid development of spectral- and image acquisition techniques, they are faced with a few drawbacks, when applied in industrial environments. Firstly, the initial establishment of specific models can be expensive and time-consuming due to laborious calibration procedures. Efforts need to be made so that the reference values can be accurately related to the relevant spectra or wavelengths of importance. In addition, a substantial amount of time, energy, and money are required for model updates. Secondly, acquisition parameters and environmental conditions can pose a significant challenge to the robustness of the model. Therefore, the data collection is influenced by the following acquisition parameters (scanning times, sample to detector distance) and environmental factors (ambient temperature, humidity, illumination conditions, sample temperature); the latter can differ in meat processing facilities. Of special note is the fact that most meat processing facilities function at low temperatures and high humidity due to food safety requirements. In order to build a robust model despite the many variable interferences, more research is still required in this area. Thirdly, another important issue is that different optical instruments lack uniformity, therefore calibration models are not interchangeable between devices. For this reason, the calibration models would have to be reconstructed which is labour-intensive and thus challenges the application. Finally, because the acquired spectral data holds large amounts of information, it is necessary to eliminate redundant features to reduce the multicollinearity problem. Various algorithms for optimal waveband selection have been developed. However, the optimal wavelengths are not consistently selected by the different methods, and the interpretability for some selected wavelengths can be rather challenging.

Despite these drawbacks, these non-destructive spectroscopic and imaging techniques have the potential to become more common in future. These methods will develop with the improvement in instrumental technology, the availability of high-speed computers, with appropriate storage capacity, and the development of appropriate chemometric procedures. Currently, the meat industry is in urgent need of an on-line/real-time detection system for the rapid authentication of meat products. The implementation of these rapid, non-destructive systems could play a vital part in the profitability of the meat industry and should thus be the main future trend. Although initial attempts achieved relative success, further research is required for the implementation of on-line systems. A number of studies claimed on-line measurements but hardly ever performed them. Hence, the literature is lacking comprehensive on-line evaluation studies and this needs to be further investigated to validate the reliability and accuracy under industrial processing conditions.

The simplification of these methods also offers the possibility to perform analysis away from the laboratory using an efficient portable, handheld, and micro-optical device. The conventional instrumentation of these methods thus has the potential to mature with the development of low-cost, miniature, fit-for-purpose, easy-to-use detection devices utilised for meat products. However, one should keep the following prerequisites in mind because both good performance hardware systems and efficient chemometrics form part of the foundation for achieving a robust model. Hence, in the near future, the research focus will shift to the development of higher sensitivity and resolution instruments and improve the stability of the implemented algorithms.

Furthermore, due to the complex meat matrix, limited information might be acquired by a single-detection method. Therefore, the combination of spectral- and imaging acquisition techniques with other emerging technologies would aid in the comprehensive evaluation of meat products by making full use of the multivariate information. In actual fact, some researchers have investigated the feasibility of combining different methods. Barbin, et al. [108] developed PLSR models to investigate the tenderness (slice shear force) prediction of pork by combining the HSI spectral information and the computer vision system’s image features. The authors concluded that the combined data improved the overall results with the model achieving a prediction *R*^2^ = 0.74. In a study by Huang, et al. [109], the authors attempted the freshness (total volatile basic nitrogen (TVB–N) content) evaluation of pork using combined NIR spectroscopy, computer vision, and electronic nose (e-nose) techniques. A backpropagation artificial neural network (BP–ANN) model was constructed for the prediction of TVB–N content and achieved an RMSEP = 2.73 mg/100 g and an *R*^2^*_p_* = 0.95. Similarly, Khulal, et al. [110] integrated HSI technology and a colorimetric sensor to predict the TVB–N content in chickens using a multiple-level data fusion (MLF) method. The BP–ANN model prediction results were improved by using the MLF approach and achieved an RMSEP = 4.31 mg/100 g and an *R*^2^*_p_* = 0.88 which was better than either of the individual system’s results. Recently, Aheto, et al. [111] investigated a multi-sensor integration approach based on HSI and e-nose for the prediction of intramuscular fat (IMF) and the peroxide value (PV) of pork. Support vector machine regression (SVMR) models were constructed for the prediction of IMF and PV. The authors performed data fusion, consequently improving the prediction results, and achieved an *R*^2^*_p_* = 0.89, 0.90 and RMSEP = 2.09, 1.01 for IMF and PV, respectively.

These integrated information studies, therefore, demonstrated the superiority of a multi-data fusion approach. By using fusion strategies, the researchers could reap the benefits by fully utilising the complementary information, particularly as pertaining to meat/meat-product quality, and in doing so overcome the shortcomings often experienced by individual sensing techniques, single feature methods, or data processing algorithms.

## 10. Conclusions

The meat industry is dynamic and complex, needing low cost and environmentally friendly, rapid techniques that ensure the quality and authenticity of products. Although most of the traditional techniques are able to detect low levels of adulteration with high reliability, they are destructive, time-consuming, and expensive. Alternatively, non-destructive techniques can provide a rapid, consistent, and objective assessment of the samples under investigation. In conjunction with appropriate multivariate data or image analysis techniques, they could be effective tools for the adulterant detection and authentication of processed meat products. The spectroscopic and imaging methods reviewed also have the potential for automation, thus eliminating tedious and time-consuming traditional methods.

Some major limitations preventing the commercial implementation of these non-destructive techniques include high-cost equipment, generation of large data sets, lengthy image processing procedures, and interpretation. Scientific knowledge is also required when selecting an efficient and practical classification algorithm, as there is no universally ideal technique; this could be a challenging task.

Future trends for meat fraud detection are moving towards developing high-performance, low-cost, and non-destructive equipment. Combining results of different non-destructive techniques, e.g., an imaging method combined with a non-imaging method could provide a more complete description of the sample under investigation. Reduction of image size and processing speed are areas of consideration if these techniques are to be used in real-time applications. Although this review mainly focussed on adulteration the technologies can also be used for chemical composition or nutritional profile and microbial contamination detection.

## Figures and Tables

**Table 1:** The advantages and disadvantages of selected non-destructive spectroscopic and imaging methods evaluated for the authentication of processed meat products.

Advantages	Disadvantages
**Vibrational Spectroscopy**	
A non-destructive technique that requires little or no sample preparation and allows for the determination of chemical and physical parameters	Hardly selective, thus chemometrics are essential to extract and decompose the analytical information for accurate interpretation
2. Rapid and affords real-time analytical information from samples	2. Difficult to obtain accurate and robust models as the construction requires large sample sets with great variations
3. Instruments are suitable for on- and in-line analysis during control procedures	3. Reference methods are required for the determination of specific parameter values
**Raman Spectroscopy**	
A non-destructive technique that requires little or no sample preparation and allows for the determination of chemical and physical parameters	Sensitive and highly optimised instruments required for detection
2. Highly specific and provides a unique fingerprint of a sample	2. Fluorescence of impurities or of the sample itself can hide the Raman spectrum
**Colour Imaging**	
A rapid, non-destructive and non-invasive technique	RGB values are not transferable between devices
2. Quantifies surface characteristics by analysing the entire surface of foodstuffs pixel-by-pixel	2. RGB models are not uniform, producing a non-linear and discontinues space
3. Requires no transformations to display data on screen	3. Challenging to identify and determine specific colours
**Hyperspectral Imaging**	
A non-destructive technique that requires no sample preparation and provides spectral and spatial information about a sample	HSI instruments are expensive, requires high hardware speed, and can be intricate
2. Provides accurate simultaneous analysis of several compounds in a single measurement	2. Significant amounts of storage space are required due to the large size of the multidimensional datasets
3. Has the potential to be implemented in an industrial set-up over a conveyer belt	3. Chemometrics is essential to extract and decompose the analytical information for accurate interpretation, thus data processing and model development is time-consuming
**X-ray Imaging and Computed Tomography**	
A non-destructive and non-invasive technique which allows for the in-depth, internal structure analysis of a sample using 3-D images	Operator dependency, cost, and time constraints as the method requires lengthy scanning and image analysis procedures

**Table 2:** A summary of selected non-destructive spectroscopic and imaging techniques and their applications for the authentication and classification of meat products.

Technique	Principle	Features	Application	Chemometric Techniques	*R*^2^ value	Reference
MIR spectroscopy	Spectral differences	Spectral features	Detection of offal adulterants in minced beef	PCA *; PLSR; CVA; SIMCA	92–100% **	Al-Jowder, et al. [55]
NIR and MIR spectroscopy			Detection of minced beef adulteration with turkey meat	PCA *; LDA; PLSR	81–99% **	Alamprese, et al. [103]
NIR spectroscopy			Detection and quantification of different adulterants in minced beef	PCA *; PLSR; LDA; PLS–DA	0.94–0.96; 100% **	Morsy and Sun [34]
Vis–NIR spectroscopy			Detection of plant and animal proteins as adulterants in minced beef and pork	PCA *; LDA; PLSR	69–100% **; 0.78–0.86	Rady and Adedeji [35]
Portable handheld NIR spectroscopy			Authentication of Iberian pork carcasses	PCA *; PLS–DA	93.9% **; 96.4% **; 60.6% **	Zamora-Rojas, et al. [104]
Portable handheld NIR spectroscopy			Classification of ‘premium’ and ‘non-premium’ Iberian hams	PCA *; LDA; QDA, NPB	1.5–2.5% error; 98% **	Piotrowski, et al. [105]
Portable handheld NIR spectroscopy			Classification of ‘premium’ and ‘non-premium’ Iberian pigs	PCA *; LDA; QDA; Kernel Bayes; Logistic regression	93.5% **	Pérez-Marín, et al. [106]
Raman spectroscopy	Provides structural fingerprints by which molecules and compounds can be identified	Spectral features	Discrimination of beef and horsemeat	PCA	100% **	Boyacı, et al. [69]
			Adulterant detection and quantification in beef burgers	PCA *; PLS–DA; SIMCA	89–100% **	Zhao, et al. [70]
MID–FTIR spectroscopy	Fourier transform algorithm turns raw data into an optical spectrum	Spectral features	Detection of adulterants in minced beef	PCA *; PLSR; SIMCA	0.99; 100% **	Meza-Márquez, et al. [8]
FT–NIR spectroscopy			Detection of pork adulteration in veal product	PCA *; SVM		Schmutzler, et al. [59]
			Detection of minced beef adulterated with turkey meat	PCA *; PLSR; PLS–DA	>0.88; 100% **	Alamprese, et al. [9]
Colour imaging	Colour variation (red, green, blue)	Hue, saturation, intensity, RGB histograms, chromaticity, coordinates, textural features	Identification and quantification of fat content adulteration in chicken burgers	PCA *; PLSR; PLS–DA; SPA–LDA	0.95; 96% **	de Sousa Fernandes, et al. [78]
			Differentiate and quantify ground chicken adulteration with pork and beef	PCA *; LDA; PLSR;	100% **; 0.82 (pork); 0.86 (beef)	Nolasco-Perez, et al. [37]
Hyperspectral imaging	Spectral and spatial differences	Spectral features and image pixels	Classification of pork, beef, and lamb	PCA *; PLS–DA	98.7% **	Kamruzzaman, et al. [33]
			Detect and quantify the adulteration level in minced lamb	PCA *; PLSR; MLR	0.99; 0.98	Kamruzzaman, et al. [89]
			Horsemeat adulteration detection in minced beef	PCA *; PLSR	0.98	Kamruzzaman, et al. [38]
			Detection of duck adulteration in minced lamb	PCA *; PLSR	0.98	Zheng, et al. [39]
			Detection of minced pork adulterated with pork jowl meat	PCA *; PLSR; 2D-COS–PLSR; PC-loadings–PLSR; RC–PLSR	0.96; 0.27; 0.74; 0.91	Jiang, et al. [90]
X-ray imaging and computed tomography	Absorption and scattering of X-ray beams	Mean grey value, grey level histogram, porosity, textural features	Detection of mechanically separated meat (MSM) in meat products			Pospiech, et al. [40]

* PCA used for exploratory statistical analysis only. ** Classification accuracy (*R*^2^ not available). PCA, principal component analysis; LDA, linear discriminant analysis; QDA, quadratic discriminant analysis; DA, discriminant analysis; BPNN, backpropagation neural network; NPB, non-parametric Bayes; CVA, canonical variate analysis; PLSR, partial least squares regression; PCR, principal component regression; MLR, multiple linear regression; PLS–DA, partial least squares discriminant analysis, SIMCA, soft independent modelling of class analogy; SVM, support vector machines; SPA, successive projections algorithm; 2D-COS, two-dimensional correction spectroscopy; PC, principal component; RC, regression coefficient.

## Data Availability

Not applicable.
